# Prognostic factors affecting the short-term efficacy of non-surgical treatment of chronic periodontitis: a multilevel modeling analysis

**DOI:** 10.1186/s40001-021-00520-y

**Published:** 2021-06-01

**Authors:** Hui-Jie Liu, Bo Wang, Ao-Chen Wang, Dan-Hong Zhang, Cui Mao, Qiu-Hong Li

**Affiliations:** 1 Department of Stomatology, The First Affiliated Hospital of Dalian Medical University, No.222 Zhongshan Road, Dalian, 116000 Liaoning China; 2 Medical Department of Graduate School, Dalian Medical University, Dalian, 116000 Liaoning China; 3 Department of Paediatric Dentistry, School of Stomatology, China Medical University, Shenyang, 110122 Liaoning China

**Keywords:** Periodontitis, Non-surgical treatment, Multilevel linear model, Prognosis

## Abstract

**Background:**

This study is aimed to analyze the prognostic factors affecting the short-term efficacy of non-surgical treatment of patients in periodontitis from stage II to stage IV by the multilevel modeling analysis.

**Materials and methods:**

A total of 58 patients with chronic periodontitis were included in this study. Patients were clinically explored before and 3 months after the treatment and the difference in probing depth was determined [Reduction of probing depth (Δ PD) = baseline PD – finial probing depth (FPD)] which is considered as the therapeutic evaluation. Three different levels were analyzed: patients, teeth and sites to construct a multi-layer linear model.

**Results:**

Probing depth (PD) improved significantly compared with that before treatment (*p* < 0.05), in which FPD was (3.90 ± 1.39) mm, and the ΔPD was (1.79 ± 0.97) mm. Compared with the mesial sites and distal sites of the multi-rooted teeth, the number of PD ≥ 5 mm or PD < 5 mm after the treatment was significantly different (*P* < 0.05), and the proportion of PD < 5 mm was higher in mesial sites. The null model showed that Δ PD varied greatly between groups at various levels (*P* < 0.001), with prediction variable of site level, tooth level, and patient level accounted for 66%, 18%, and 16% of the overall difference, respectively. The complete model showed that the Δ PD of smokers was significantly lower than that of non-smokers (*P* < 0.001). The Δ PD of the mesial and distal sites was larger than that of the buccolingual central site (*P* < 0.001). The Δ PD of single-rooted teeth was larger than that of multi-rooted teeth (*P* < 0.001). The baseline PD, tooth mobility (TM), bleeding index (BI), clinical attachment loss (CAL) were significantly negatively correlated with Δ PD (*P* < 0.001).

**Conclusions:**

Patients with periodontitis from stage II to stage IV, who were non-smoking, have good compliance, good awareness of oral health, and low percentage sites with PD ≥ 5 mm at baseline, single-rooted teeth with hypomobility, less clinical attachment loss and lower bleeding index and sites of mesial or distal can obtain an ideal short-term efficacy of non-surgical treatment.

**Supplementary Information:**

The online version contains supplementary material available at 10.1186/s40001-021-00520-y.

## Background

As a chronic disease, the risk factors that have been identified for periodontitis include genetics, systemic diseases, lifestyle, sociological and environmental factors [1, 2]. Meanwhile, jointly determined by the American Academy of Periodontology (AAP) and the European Federation of Periodontology (EFP), periodontitis was, divided into four stages (I–IV) and three levels (A/B/C), which also reflects the current focus of scholars on judging the progress of individual periodontitis by combining risk factors[3]. The combination of various risk factors not only makes the state of periodontitis and the progression vary in different individual, but also leads to great differences in treatment response.

Periodontal non-surgical treatment is a process of thoroughly removing the biofilm and calculus on the tooth root surface to reduce the total amount of bacteria below the individual disease threshold level, which has been proved its overall efficacy by previous studies [4, 5]. Meanwhile, it is necessary to integrate various factors to speculate whether the patients will respond well to the non-surgical treatment.

A traditional logistic regression or the analysis of variance was often used to process periodontal data, which ignores the three different levels involved in periodontal data: patients, teeth and sites. It will underestimate the standard errors and give potentially misleading results when the data at the lower level are averaged to the higher level. Hierarchical linear model (HLM) can be used to process multilevel nested data. It can not only decompose the variation at different levels, but also analyze the interaction between different levels to accurately separate the effect of prediction variables at all levels. The HLM has been widely used in periodontal studies since first proposed by Sterne et al. [6] in 1988. Also, Albandar et al. [7] have applied HLM to study the predictors of periodontal disease progress at the level of patient individuals and teeth position. Vettore et al. [8] used this method to analyze the relationship between the prevalence of periodontal disease, geographical location and social status. Furthermore, the HLM has also been applied in processing the nested data of prosthodontics and orthodontics [9, 10]. This study was designed to analyze the prognostic factors affecting the short-term efficacy of non-surgical treatment of chronic periodontitis patients by the method of HLM.

## Materials and methods

### Patients

The study was approved by the Ethics Committee of the First Affiliated Hospital of Dalian Medical University and the informed consent forms were signed by the involved individuals. Patients from the Stomatology of The First Affiliated Hospital of Dalian Medical University from June 2018 to June 2019 were included in this study. A questionnaire was distributed among all the patients to obtain personal information (Additional file 1: Table S1). The inclusion criteria included: (1) patients with clinically stage II to stage IV periodontitis (Classification standard in Additional file 1: Table S2) [3]; (2) aged between 18 and 80; (3) at least 16 teeth remained (excluding the third molar). The exclusion criteria included: (1) refusal of non-surgical periodontal therapy; (2) basic periodontal treatment history and periodontal surgery history; (3) antibiotics usage within one month; (4) pregnancy and lactation; (5) systemic disease history; (6) serious mental illness and cognitive impairment.

### Clinical data collection

**Fig. 1:**
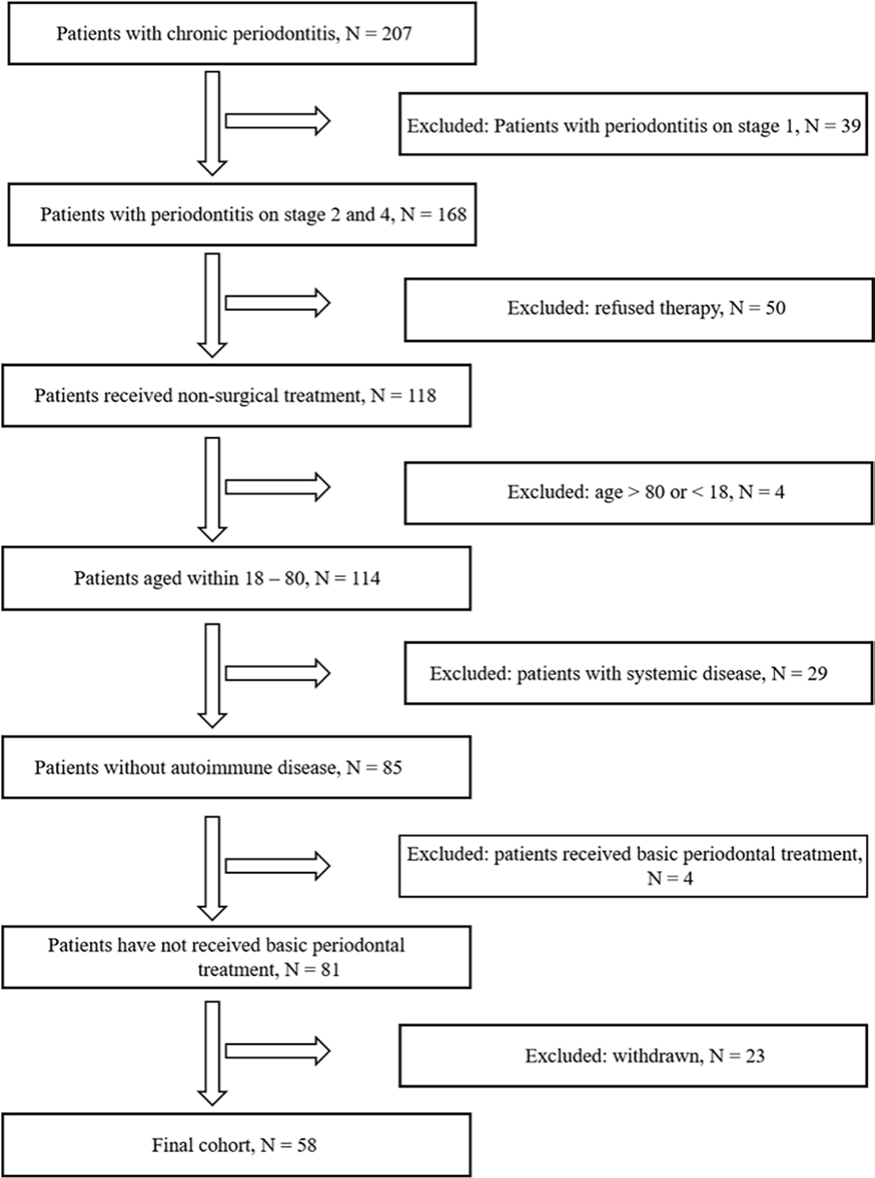
The inclusion flowchart of patients in this study

The demographic data of the patients included in the study were recorded, and the patients were treated with non-surgical periodontal treatment. A full-mouth set of radiographs were obtained and clinical examinations were performed before treatment. The whole teeth were divided into four quadrants (upper left, lower left, upper right and lower right). Six sites (mesio-buccal, disto-buccal, mid-buccal, mesio-lingual, disto-buccal, mid-lingual) per tooth were measured and recorded, excluding the third molars. Probing depth (PD), clinical attachment loss (CAL), bleeding index (BI), tooth mobility (TM) were probed with a periodontal probe. After the treatment, oral hygiene instruction regarding brushing and inter-dental cleaning, and regular follow-up visits at 2 weeks, 1 and 3 months were informed. There was no treatment during the follow-up visits. Among them, follow-up visits in 2 weeks and 1 month were to observe patient compliance, such as the degree of plaque control, dental floss and interdental brush usage. Meanwhile, oral hygiene education was instituted to ensure that all patients strengthen their oral health awareness at the same time. Finial probing depth (FPD) was measured in the 3-month follow-up visits. Patients who could not be observed during the 3 months were excluded. According to the inclusion flowchart in Fig. 1, 58 patients were ultimately included in this study. All the above procedures were carried out by two experienced periodontists, who were trained to adequate levels of accuracy and reproducibility for the various clinical parameters and indices to be used. The repeatability test showed that the Kappa value of each index was > 0.75, which proved that the consistency was good.

The collected data were divided into three levels, including site level, tooth level and patient level.

Therein, the site level included: (1) baseline PD; (2) site location (mesio-buccal, disto-buccal, mid-buccal, mesio-lingual, disto-lingual, mid-lingual).

The tooth level included: (1) the BI of the tooth was detected for 30 s (the BI of tooth with higher buccal and lingual surface was recorded as the BI of tooth) (0–5); (2) the TM of the tooth (0–III°); (3) CAL: measured by the distance from the cemento-enamel junction to the bottom of the periodontal pocket (the maximum CAL of buccal and lingual surface of each tooth was recorded, and the mean CAL of buccal and lingual was calculated); (4) tooth type: single-rooted and multi-rooted.

The patient level included: (1) age; (2) gender; (3) smoking status; (4) body mass index (BMI); (5) compliance: according to the follow-up situation, the patients were divided into the complete compliance group (patients who participate in each follow-up on time, and perform well in brushing and inter-dental cleaning) and irregular compliance group (absent during the follow-up or/and occasionally use floss or interdental brush); (6) education: high school degree or below, bachelor's degree and postgraduate degree or above; (7) oral health awareness (OHA) score: a questionnaire contains four questions about OHA of patients, the answers were counted as very important, generally important and unimportant, each of which was scored as 3, 2 and 1, respectively. Each patient obtained a minimum score of 4, and a maximum score of 12, which was recorded as the OHA score [11]; (8) the percentage sites with PD ≥ 5 mm at baseline.

### Statistical analysis

The sites with baseline PD < 5 mm can obtain considerable outcomes after the periodontal non-surgical treatment. Hence, the object of this study was all sites with a baseline PD ≥ 5 mm, and define the HLM outcome variable (ΔPD = baseline PD–FPD). The HLM is composed of fixed effect and random effect. Therein, the value of fixed effect refers to the regression coefficient (the average slope and intercept), and the value of random effect refers to the variance of the residual (the specific part of different groups). Moreover, HLM analysis starts with a null model. The null model means that no variables are added to the model. This mode is used to judge whether multilevel modeling was required by estimating whether the overall difference of variables was significant and attribute it to the patient, tooth, and site levels [12]. Then, according to the purpose of the study, the random effects model and the mixed-effects model are constructed by adding the prediction variables of each level. Some studies used the random intercept model, which only considered the change of the intercept between each level, rather than that of the slope [13, 14]. However, Litière has suggested that consider the change of the slope in each level will improve the accuracy of the model [15]. At the same time, Müller found that the random effects model can better describe the actual situation than the random intercept model [16]. Hence, we use the random effects model, which considers whether there is a difference in slope between different groups by randomizing all variables that may have random effects. Instead of random intercept model, we use the mixed-effects model, which not only contains all the variables in the three levels, but also considers the change in slopes between different levels. It can explain how the dependent variable is affected by each level better.

All data were manifested as count (percentage) or mean ± standard deviation (SD) and were imported into SPSS 22.0 software (IBM Corp., Armonk, NY, USA), and HLM 6.0 software (Scientific Software International Inc., Lincolnwood, USA) for analysis. The comparison of continuous measurement data was analyzed by Student’s t test of paired samples, the comparison of counting data was analyzed by Pearson x^2^ test, and the nested structure data was analyzed by a multi-layer linear model method. A *P* value < 0.05 was regarded as statistically significant.

## Results

### Demographic data and variables assignment

Table 1 reveals the demographic data and variables assignment of this study. A total of 58 patients, 1162 teeth and 3126 sites were included in this study.

**Table 1:** The demographic data and variables assignment of this study

Variables	Assignment	Mean ± standard deviation (SD)
Patient level (third level)		N = 58
Age, years	Continuity variable	45.00 ± 12.56
Gender	Male = 1/female = 2	34/24
Smoking	Smoking = 0/non-smoking = 1	40/18
BMI, kg/m^2^	Continuity variable	24.97 ± 2.57
Compliance	Complete compliance = 1/irregular compliance = 2	32/26
Percentage of baseline PD ≥ 5 mm	Continuity variable	34.75 ± 16.24
OHA score, point	Continuity variable	6.52 ± 1.23
Education	High school degree or below = 1/bachelor's degree = 2/postgraduate degree or above = 3	27/24/7
Tooth level (second level)		N = 1162
Baseline BI	0/1/2/3/4/5	0/102/342/450/268/0
Tooth type	Single tooth = 1/multiple teeth = 2	708/454
TM, degree	0 degree = 0/I degree = 1/II degree = 2/III degree = 3	936/138/71/17
CAL, mm	Continuity variable	4.26 ± 1.22
Site level (first level)		N = 3126
Baseline PD	Continuity variable	5.69 ± 1.08
Site location	Mesio-buccal, disto-buccal, mesio-lingual, disto-lingual sites = 1/mid-buccal, mid-lingual site = 2	2714/412

*BMI* body mass index, *PD* probing depth, *OHA* oral health awareness, *BI* bleeding index, *TM* tooth mobility, *CAL* clinical attachment loss

### Description and comparison of PD at different sites before and after treatment

**Fig. 2:**
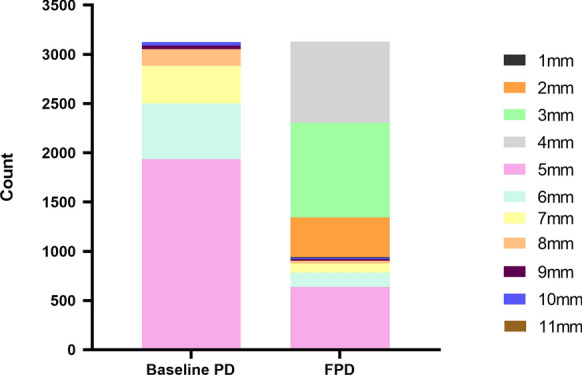
Proportion of PD before and after non-surgical treatment

At the 3-month follow-up visit, PD improved significantly compared with that before treatment (*p* < 0.05), in which FPD was (3.90 ± 1.39) mm, and the ΔPD was (1.79 ± 0.97) mm (Table 2). Besides, according to the proportion of PD before and after treatment, FPD was mainly distributed in 3-5 mm, which accounted for 30.7% (960/3126), 26.4% (825/3126), and 20.4% (637/3126), respectively (Fig. 2). Among them, there were 1739 sites with PD ≥ 5 mm in single-rooted teeth and 1387 sites with PD ≥ 5 mm in multi-rooted teeth at baseline. After 3 months of non-surgical periodontal treatment, 1341 sites PD < 5 mm and 398 sites PD ≥ 5 mm were found in single-rooted. There were 865 sites with PD < 5 mm and 522 sites with PD ≥ 5 mm in multi-rooted. With PD ≥ 5 mm or PD < 5 mm as the boundary, the number of 6 sites of single-rooted teeth and multi-rooted teeth were compared in pairs. The results showed that in multi-rooted teeth (Table 3), there was a significant difference in the number of sites with PD ≥ 5 mm or PD < 5 mm after treatment between mesio-buccal and disto-buccal (*P* < 0.05), and there was a higher proportion of PD < 5 mm in the mesio-buccal. Moreover, there was a significant difference in the number of sites with PD ≥ 5 mm or PD < 5 mm after treatment between the mesio-lingual and disto-lingual (*P* < 0.05). The proportion of PD < 5 mm in the mesio-lingua was higher, and there was no statistical difference in the rest. In single-rooted teeth (Table 4), there was no statistical difference in the number of sites with PD ≥ 5 mm or PD < 5 mm at 6 sites after the treatment.

**Table 2:** PD changes before and after treatment (mm)

	Baseline	FPD	ΔPD	*P* value
PD (mean ± se)	5.69 ± 1.08	3.90 ± 1.39	1.79 ± 0.97	0.00^*^

*PD* probing depth

*FPD* finial probing depth

*P < 0.05

**Table 3:** Comparison of PD ≥ 5 mm or < 5 mm at each site after 3 months of multi-rooted teeth treatment

	PD < 5 mm		PD ≥ 5 mm		Total	*χ*^2^	*P* value
	Number of sites	Constituent ratio (%)	Number of sites	Constituent ratio (%)			
Mesio-buccal site	203	75.5	66	24.5	269	1.11	0.29
Mesio-lingual site	206	71.5	82	28.5	288		
Mesio-buccal site	203	75.5	66	24.5	269	23.37	0.00*
Disto-buccal site	166	56.1	130	43.9	296		
Disto-buccal site	166	56.1	130	43.9	296	0.11	0.74
Disto-lingual site	182	57.4	135	42.6	317		
Mesio-lingual site	206	71.5	82	28.5	288	13.07	0.00*
Disto-lingual site	182	57.4	135	42.6	317		
Mid-buccal site	49	47.	54	52.4	103	0.38	0.54
Mid-lingual site	59	51.8%	55	48.2	114		

*PD* probing depth. *P value < 0.05

**Table 4:** Comparison of PD ≥ 5 mm or < 5 mm at each site after 3 months of single-rooted teeth treatment

	PD < 5 mm		PD ≥ 5 mm		Total	*χ*^2^	*P* value
	Number of sites	Constituen*t* ratio (%)	Number of sites	Constituen*t* ratio (%)			
Mesio-buccal site	303	77.3	89	22.7	392	0.15	0.72
Mesio-lingual site	277	78.5	76	21.5	353		
Mesio-buccal site	303	77.3	89	22.7	392	0.16	0.69
Disto-buccal site	324	78.5	89	21.5	413		
Disto-buccal site	324	78.5	89	21.5	413	0.22	0.64
Disto-lingual site	299	64.8	89	35.2	388		
Mesio-lingual site	277	78.5	76	21.5	353	0.21	0.65
Disto-lingual site	299	64.8	89	35.2	388		
Mid-buccal site	49	77.8	14	22.2	63	1.81	0.18
Mid-lingual site	89	68.5	41	31.5	130		

*PD* probing depth

### Multilevel modeling analysis

1. The null model
  The results of the null model without any predictive variables (Table 5) showed that significant variations existed in all three levels (*P* < 0.001), which was necessary to build a multilevel linear model to explain the difference. In the null model variability at each level was obtained as percentage of the total variability calculated adding all estimates together. Therein, the majority of the variance in Δ PD was attributed to the site level (66%), followed by the tooth (18%) and patient levels (16%).
2. The random effects model
  The results of the random effects model (Table 6), showed that the test for the intercept item at level 3 was significant (*P* < 0.001), indicating that it was necessary to continue to include the variables of level 3 in the intercept item to explain differences. Hence, we construct the random effects model, which considered whether there was a difference in slope between different groups by randomizing all variables that may have random effects. The random part of the baseline PD μ_2_ slope was not significant (*P* > 0.05), then discarded the random slope and set it as a fixed effect. Meanwhile, independent variable slopes in TM, BI, CAL, and the baseline PD e_20_ were significantly different (*P* < 0.001), and needed to be randomized in the mixed-effects model.
3. The mixed-effects model
  All the clinical covariates of three levels were added to the model to build a mixed-effects model (Table 7), which also contain the change in slopes between different levels. At the site level, the model confirmed that mesial and distal sites were the areas where PD changes were greater than those observed at the buccolingual central site (*P* < 0.001). Meanwhile, the deeper the baseline PD, the greater the observed decrease in PD after non-surgical periodontal treatment (*P* < 0.001). At the tooth level, compared with multi-rooted teeth, the Δ PD of single-rooted teeth after periodontal non-surgical treatment was higher (*P* < 0.001). Meanwhile, there was a significant negative correlation between BI, TM, CAL and Δ PD at the baseline (*P* < 0.001). At the patient level, compared with the non-smokers, smoking had a significant negative impact on the chance of PD (*P* < 0.001). Δ PD was significantly affected by patient compliance (*P* < 0.01), and the Δ PD of patients with complete compliance was higher than that with irregular compliance. The greater the percentage sites with PD ≥ 5 mm at baseline, that is, the patients with the poor initial full periodontal conditions, the ΔPD was relatively small (*P* < 0.01). In addition, the OHA score (Additional file 1: Table S3) was positively correlated with the oral health awareness of patients and Δ PD *(P* < 0.01). However, there was no significant correlation between age, gender, education level and BMI (*P* > 0.05).

**Table 5:** The estimation results of null model

	Standard deviation	Variance component	Chi-square value	*P* value
Random effect part				
Site level (first level)	0.79	0.62 (66%)		
Tooth level (second level)	0.41	0.17 (18%)	1904	0.000^***^
Patient level (third level)	0.38	0.15 (16%)	453	0.000^***^
− 2logL			8089.56	

***P value < 0.001

*− 2logL* − 2 times the log likelihood

**Table 6:** The random effects model

	Variance component	Chi-square value	*P* value
Random effect part			
Intercept μ_00_	0.09	310.76	0.000***
Baseline PD μ_2_	0.01	317.49	> 0.5
Intercept e_000_	0.89	82.36	0.000***
TM	0.05	103.51	0.000***
BI	0.01	52.22	0.024*
CAL	0.01	65.62	0.001**
Baseline PD e_20_	0.03	123.24	0.000***

***P < 0.001, **P < 0.01, *P < 0.05

**Table 7:** The mixed-effects model

	Coefficient	Standard deviation	Variance component	Inspection value	*P* value
Fixed effect part					
Intercept	2.45	0.41		6.02^a^	0.000***
Site location	− 0.3	0.05		− 6.03^a^	0.000***
Baseline PD	0.30	0.03		9.60^a^	0.000***
Tooth type	− 0.39	0.04		− 8.82^a^	0.000***
TM	− 0.32	0.04		− 7.34^a^	0.000***
BI	− 0.20	0.03		− 7.17^a^	0.000***
CAL	− 0.14	0.02		− 5.93^a^	0.000***
Age	0.001	0.004		0.34^a^	0.739
Gender	− 0.09	0.07		− 1.30^a^	0.199
Smoking	− 0.35	0.08		− 4.36^a^	0.000***
Compliance	− 0.21	0.07		− 3.14^a^	0.003**
BMI	− 0.01	0.01		− 0.91^a^	0.365
Percentage of baseline PD ≥ 5 mm	0.005	0.002		2.50^a^	0.016**
OHA score	0.07	0.03		2.35^a^	0.023**
Education	0.03	0.06		0.45^a^	0.658
Random effect part					
Intercept μ_00_		0.14	0.02	611.03^b^	0.047*
Intercept e_000_		0.96	0.93	94.36^b^	0.000***
BI		0.13	0.02	51.28^b^	0.029**
TM		0.22	0.05	104.47^b^	0.000***
CAL		0.10	0.01	64.87^b^	0.001**
Baseline PD e_20_		0.18	0.03	129.10^b^	0.000***
− 2logL	7176.81			912.75^c^	0.000***

*BMI* body mass index, *PD* probing depth, *OHA* oral health awareness, *BI* bleeding index, *TM* tooth mobility, *CAL* clinical attachment loss, − 2logL, − 2 times the log likelihood

***P < 0.001, **P < 0.01, *P < 0.05

^a^*t* value; ^b^*x*^2^ value; ^c^*x*^2^ value compared with the − 2logL goodness of fit of the null model

From the random effects of the complete model, there was a significant random effects in BI, TM, CAL and baseline PD (*P* < 0.05, *P* < 0.001, *P* < 0.01), indicating that these factors are still affected by other clinical covariates, which can be further studied. Meanwhile, − 2 times the log likelihood (− 2logL) of the mixed-effects model is improved significantly compared with the null model (7176.81 to 8089.56, *P* < 0.001), which suggested that mixed-effects model and these predictor variables were significantly fit to the data [12].

## Discussion

Currently, it is generally acknowledged that the PD ≥ 5 mm after 3 months of periodontal non-surgical treatment indicates that subsequent treatment is needed. Regarding this, Tomasi et al. [17] have put forward the concept of “periodontal pockets closure”, that is, “PD < 5 mm” as a clinical endpoint to be evaluated after 3 months of periodontal treatment. However, for many patients with moderate-to-severe periodontitis, the deep periodontal pockets perform obvious effect even though FPD > 5 mm after non-surgical treatment, shows that the concept of “periodontal pockets closure” is not fully consistent with the efficacy of non-surgical periodontal treatment. Hence, in this study, Δ PD and the site of baseline PD ≥ 5 mm were used to act as the result variable and the study object of the model, respectively.

The results of null model showed that the ΔPD was varied significantly among the site level, tooth level and patient level, and they accounted for 66%, 18%, and 16% of the overall variations respectively. It indicated that the factors in site level mattered most on short-term efficacy of periodontal non-surgical treatment. The results of mixed-effects model showed that the mesial and distal sites showed significantly greater changes in PD after non-surgical periodontal therapy than buccolingual central site at the site level, which was similar to previous studies by D’Aiuto et al. [18]. In addition, compared with the average reduction of 1.79 mm in PD after treatment, other studies have also found that after non-surgical treatment of medium-depth periodontal pockets, the ΔPD was 1.2 mm, and those of deep periodontal pockets (PD ≥ 6 mm), the PD decreased by an average of 2.4 mm [19]. Similarly, this study showed a positive correlation between Δ PD and baseline depth, which may be related to the control of inflammation after the treatment, the swollen gums subsided obviously, and the long epithelium formed on the root surface, which made the decrease of PD in deep periodontal pockets more obvious than in shallow periodontal pockets.

At the tooth level, the Δ PD of multi-rooted teeth was smaller than that of single-rooted teeth, which can be explained from three aspects. Firstly, single-rooted teeth were mainly located in the front of the dentition, with high treatment efficiency, and the gingival tissue in the anterior teeth area was thinner than that in the premolars or molars, with a better healing degree. Secondly, the anatomical structure of multi-rooted teeth was complicated because of complex root canal system, the presence of furcations, enamel pearl and root depression. Shi et al. [20] found that the furcation involvement dramatically affected the reduction of PD in the molars, which tend to obtain the poor prognosis [21]. Thirdly, the multi-rooted teeth often bear greater masticatory force than the single-rooted teeth. This model also demonstrated that teeth with severe CAL and hypermobility were associated with unsatisfying prognosis, which indicated that the effect of non-surgical treatment on hopeless teeth or questionable teeth is limited and surgical treatment or extraction should be involved to achieve a better outcome.

Smoking has been proved to be one of the main risk factors for the occurrence and development of periodontal diseases. Compared with non-smokers, smokers have deeper periodontal pockets and higher CAL, more obvious alveolar bone resorption, more severe gingival recession, and higher risk of teeth loss [22]. In this study, cigarette smoking negatively affects the outcome of non-surgical periodontal therapy: smokers had 0.35 mm less ΔPD than non-smokers, which was similar to the previous study by Bunaes et al. [23]. This may be related to the fact that the depth of periodontal pockets and the level of CAL of smokers were larger than that of non-smokers, and the treatment difficulty was correspondingly increased. In addition, because of the lower degree of tissue inflammation in smokers, during the process of periodontal exploration, the probe penetration was reduced, and the measured CAL was underestimated. Moreover, the ecological environment in deep periodontal pockets of smokers was more difficult to improve by simple mechanical debridement. Regarding this, the previous studies have showed that after 3 months of periodontal treatment, a significant reduction in red and orange complexes was only observed in non-smokers. After 6 months of treatment, subcolonial bacterial recolonization of pathogenic bacteria was observed only in smokers, indicating that smokers were more likely to reconstruct pathogenic subgingival plaque biofilms than non-smokers [24].

Furthermore, compliance is another important factor that affected periodontal non-surgical treatment. Although the compliance grouping methods reported in the literature typically varied, the registered attendance rate was consistantly regarded as the grouping principle [25]. In addition, we also considered the patient’s compliance with the physician’s oral hygiene guidelines, such as the degree of plaque control, dental floss and interdental brush usage. After 3 months of treatment, patients in the complete compliance group had significantly higher Δ PD than those in the irregular compliance group, which demonstrated that good compliance is essential to a successful periodontal treatment. Besides, the awareness of plaque control among patients with painless periodontitis decreased after the treatment. And they would withdraw from the follow-up until the dysfunction such as more severe bleeding gums and hypermobility in teeth occurred. Lee et al. [26] found that patients with complete compliance had significantly lower rates of tooth loss during supportive periodontal therapy (SPT) than patients with irregular compliance. Moreover, Robinson et al. [27] and Furuta et al. [28] have found that women had a stronger anti-infection ability, healthier oral habits and better overall prognosis of periodontal treatment than that of men [29]. However, in this study, the factors of age and gender showed no significant correlation, which may be related to the limited sample size. Meanwhile, − 2logL of the final model was significantly different from that of the null model, which indicated that the difference was significantly improved after all covariates were added to the model, but there were still unexplained parts. Hence, variables can be added to future studies to further explain the model, such as the factors of furcation involvement, pulp status, crown root ratio, direction of bone resorption, crowded degree of dentition, width of attached gingiva, subgingival plaque microorganisms, systemic diseases, and genetics.

The enlightenment of this study for clinicians is that the prognosis of periodontal non-surgical treatment for periodontitis patients with smoking, poor compliance and poor awareness of oral health may be poor. The clinicians should emphasize the necessities of quitting smoking and SPT, strengthen oral hygiene instruction for such patients. Moreover, the multi-rooted teeth, teeth with severe TM, BI and CAL often suggest lower PD reduction, especially the buccolingual central site, which requires clinicians to fully consider the above factors when making the treatment plans for initial diagnosis. It is also necessary to focus on these teeth and sites during the long-term follow-up to prevent further disease progression.

## Conclusions

Patients with periodontitis from stage II to stage IV, who were non-smoking, have good compliance, good awareness of oral health, and low percentage sites with PD ≥ 5 mm at baseline, single-rooted teeth with hypomobility, less CAL and lower BI and sites of mesial or distal tend to obtain a more ideal short-term efficacy of non-surgical treatment.

## Supplementary Information

**Additional file 1.** **Table S1.** Questionnaire of the chronic periodontitis patients. **Table S2.** New stages of periodontitis [3]. **Table S3.** Question-wise mean of oral health awareness scores.

## Data Availability

The datasets used and/or analyzed during the current study are available from the corresponding author on reasonable request.

## Abbreviations

PD: Probing depth
FPD: Finial probing depth
TM: Tooth mobility
BI: Bleeding index
CAL: Clinical attachment loss
AAP: American Academy of Periodontology
EFP: European Federation of Periodontology
HLM: Hierarchical linear model
BMI: Body mass index
OHA: Oral health awareness
SPT: Supportive periodontal therapy
