# Supplementary material for: Prognostic factors affecting the short-term efficacy of non-surgical treatment of chronic periodontitis: a multilevel modeling analysis
Source: Eur J Med Res. 2021 Jun 1;26:50. doi: 10.1186/s40001-021-00520-y (PMC8170789; doi:10.1186/s40001-021-00520-y)
Supplement: Supplementary file 1 — Additional file 1. Table S1. Questionnaire of the chronic periodontitis patients. Table S2. New stages of periodontitis [3]. Table S3. Question-wise mean of oral health awareness scores. [file 40001_2021_520_MOESM1_ESM.docx]

Table S1: Questionnaire of the chronic periodontitis patients

Code：

Treatment time：

Diagnosis：Periodontitis is in the _____ stage

Whether you have received periodontal basic treatment:

🞎Yes (The recipient continues to answer the following eight questions)

🞎No

General situation of patients

1. Name：__________
2. Age：__________
3. Gender：

🞎Male

🞎Female

1. Education：

🞎 High school degree or below

🞎 Bachelor's degree

🞎 Postgraduate degree or above

1. Smoking：

🞎Yes

🞎No

1. Height：________
2. Weight：________
3. Tel：___________
4. Basic clinical information of patients
5. Whether you have a systemic disease：

🞎 Hypertension

🞎 Diabetes

🞎 Cardio cerebrovascular disease

🞎 Nephropathy

🞎 Hepatitis

🞎 Hematopathy

🞎 Others：__________

1. Whether you in the pregnancy or lactation：

🞎Yes

🞎No

1. Periodontal treatment history
2. Whether you have received periodontal basic treatment

🞎Yes

🞎No

1. Whether you have received periodontal surgical treatment

🞎Yes

🞎No

1. Oral health awareness (Note: to be completed with the assistance of clinicians)
2. Importance of dental health to overall health：

🞎Very important

🞎 Medium importance

🞎 Not very important

1. Importance of tooth brushing：

🞎 Very important

🞎 Medium importance

🞎 Not very important

1. Importance of interdental space cleaning：

🞎 Very important

🞎 Medium importance

🞎 Not very important

1. Your concern level of gum disease：

🞎 Very important

🞎 Medium importance

🞎 Not very important

Table S2: New stages of periodontitis[3]

| Periodontitis stage | Severity | | | Complexity | Extent and distribution |
| --- | --- | --- | --- | --- | --- |
|  | Interdental CAL | Radiographic bone loss | Tooth loss | Local | Add to stage as descriptor |
| Stage I  Stage II  Stage III  Stage IV | 1 to 2 mm  3 to 4 mm  ≥5 mm  ≥5 mm | Coronal third (<15%)  Coronal third (15% to 33%)  Extending to mid-third of root and beyond  Extending to mid-third of root and beyond | No tooth loss due to periodontitis  Tooth loss due to periodontitis of ≤4 teeth  Tooth loss due to periodontitis of ≥5 teeth | Maximum probing depth ≤4 mm Mostly horizontal bone loss  Maximum probing depth ≤5 mm Mostly horizontal bone loss  In addition to stage II complexity:  Probing depth ≥6 mm Vertical bone loss ≥3 mm  Furcation involvement Class II or III Moderate ridge defect  In addition to stage III complexity:  Need for complex rehabilitation due to: Masticatory dysfunction Secondary occlusal trauma (tooth mobility degree ≥2)  Severe ridge defect  Bite collapse, drifting, flaring Less than 20 remaining teeth (10 opposing pairs) | For each stage, describe extent as localized (＜30% of teeth involved)，generalized, or molar/incisor pattern |

Table S3: Question-wise mean of oral health awareness scores

| Question number | Question | Mean of OHA score |
| --- | --- | --- |
| 1 | Importance of dental health to overall health | 1.6 |
| 2 | Importance of tooth brushing | 2.1 |
| 3 | Importance of interdental space cleaning | 1.4 |
| 4 | Your concern level of gum disease | 1.4 |

Note: OHA, oral health awareness
